# African leadership for sustainable health policy and systems research

**DOI:** 10.1186/1472-6963-13-S2-S15

**Published:** 2013-05-31

**Authors:** Cheikh Seydil Moctar Mbacke

**Affiliations:** 1 Independent Consultant, Dakar, Senegal

## Introduction

The African Health Initiative (AHI) is the Doris Duke Foundation’s (DDCF) response to the need to improve the knowledge base on how to strengthen health systems. As this Initiative began, African leaders already had set the stage for a focus on the health system through a series of high level meetings, all with accompanying declarations, beginning in 2004. African leadership, in committing governments to strengthening health systems, seemed likely to support AHI efforts to improve population health in ways that could be **sustained** by governments in the focus countries after the program ends. Elements of the program designed to assure sustainability included alignment with national and local health priorities, ownership and participation by the national Ministry of Health and affected communities, building of local capacity, and effective African leadership. But, as is often the case, challenges to African leadership and sustainability continue to emerge. This comment reviews progress and prospects in achieving sustainability. I argue that sustainability lies at the core of country ownership and will require a reshaping of how both countries and funders engage to improve population health.

## Emergence of an African perspective on health systems strengthening

The “era of health systems strengthening” can be dated to a ministerial summit held in Mexico City in 2004, which resulted in the Mexico Statement on Health Research and signaled the emergence a new era for global health. It called on national governments to commit more funding to research on health systems and other funders of health research to “support a substantive and sustainable program of health systems research aligned with priority country needs” [1].

This call came shortly after the launch of large-scale disease-specific initiatives to address HIV, malaria, and tuberculosis, and just as global mobilization began in earnest to address the Millennium Development Goals (MDGs). At least three major factors aligned to drive a shift in attention and resources to health systems strengthening: realization that weak health systems impede the achievement of health MDGs, growing evidence on the adverse effects of global health initiatives on national health systems, and the recognition by global health initiatives that strong health systems were critical to the achievement of their organizational goals [2].

The shift occurred despite the absence of a solid body of evidence supporting the theory and practice of health systems strengthening and Africa was at the center stage of this development. Addressing the knowledge gap led to a growing call for health-related research into operations, systems, policy and implementation issues, highlighted nearly a decade and a half previously in the work of the Commission on Health Research for Development. Between 2006 and 2008 alone, six high-level meetings were held in Africa that led to declarations of commitment and call to action for health systems strengthening: Abuja, Nigeria (March 2006); Accra, Ghana (June 2006); Johannesburg, South Africa (April 2007); Ouagadougou, Burkina Faso (April 2008); Algiers, Algeria (June 2008); and Bamako, Mali (November 2008). In addition to building member country buy-in for an emerging agenda, this intensive schedule attempted to escalate investment in health research — a pathway to evidence-based strategies. These African meetings helped shape the current African vision for health and the World Health Organization strategy for research on health, which identifies health systems strengthening as a top priority [3].

Recognition of the growing role of research increased over time. At the emergence of the global consensus on health systems strengthening, the dominant state of mind is embodied by the African Regional Health Report of 2006 [4]. While recognizing the critical need to strengthen health systems, the only type of research envisaged in the report was “research and development to find more effective medicines and vaccines!” But this position evolved extremely quickly and later was broadened substantially at two high-level ministerial meetings on health research held in Abuja in March 2006 and in Accra three months later. The stated purpose of the Abuja meeting was explicitly to “develop an **African perspective** on health research for achieving and sustaining health MDGs in the African continent.” These two meetings recognized the existing knowledge gaps in the performance of health systems and the critical need for health systems research in providing evidence-informed strategies for health policy and systems strengthening. Acknowledging the role of non-biomedical health -related research was a large step. Convinced that African governments should provide coordinated leadership and influence regional and global health research agendas, the participants committed to developing a comprehensive national health research policy framework by 2007.

The African vision for health research was nailed down in the “African Health Strategy 2007-15” that was approved by the Third Session of the African Union Conference of Ministers of Health held in Johannesburg in April 2007 [5]. The strategy is an attempt to harmonize the existing health strategies and to provide an “inspirational framework” for the Africa Union, member States and Regional Economic Communities. It reaffirms the focus on health systems strengthening with the aim to better reach the poor, those most in need of health care and to reduce poverty. It also declared the centrality of research to guide this effort, so that what works would be distinguished from what doesn’t, thereby assuring that scarce resources were well spent. The strategy explicitly calls on countries to build research capacity and invest at least 2% of national health expenditure and 5% of aid funds in research that contributes to improving the performance of their health systems.

Ouagadougou (April 2008) and Algiers (June 2008) were a prelude to the Global Ministerial Forum on research for Health which was held for the first time in Africa, Bamako 17-19 November 2008. The Ouagadougou meeting on primary health care and health systems in Africa took stock of lessons learnt in the implementation of primary health care (PHC) since Alma Ata (September 1978) and urged countries to rekindle the Primary Health Care approach with a view to strengthening health systems to achieve the health MDGs. The Algiers Ministerial Conference on Research for Health in the Africa Region (23-25 June 2008) reaffirmed the critical role of research and called on countries to take the driver’s seat by identifying their own health priorities and investing in the development of strong national health research systems. Algiers provided an opportunity to agree on a common African declaration for submission to the Bamako Forum.

## Putting the money where our mouth is

Because Africa is burdened by widespread dysfunction of its health systems, it was encouraging that its leadership rose to the challenge and played an important role in moving forward such a research agenda. But the move from proclamation to action has been undeniably slow. It took an awfully long time since the report of the Commission on Health Research for Development in 1990 [6] for African countries to recognize and embrace research on health systems as a fundamental ingredient in their efforts to improve health in a sustainable way.

Furthermore, it is disheartening that the constant in all the African high-level ministerial meetings remains the recommendation made almost a quarter-century ago by the Commission on Health Research for Development that low and middle income countries should allocate at least 2% of national health expenditures and 5% of externally funded programs to research and research capacity strengthening. Despite the many calls to action during the last decade, African governments are still dragging their feet in fulfilling these commitments, as they have on the commitment made in Abuja to invest 15% of their annual budgets in health. It is estimated that by 2011 (10 years after the Abuja Declaration), only one country had reached that 15% target, and that the share of government budgets that went to health decreased, or, at best, remained stagnant in 20 countries or 38% of the African Union membership [7]. Sharing the failure to keep commitments are the rich countries, whose targets for development of assistance go unmentioned and unmet. Donor countries are not delivering on their commitments to allocate 0.7% of their gross national product to Official Development Assistance (ODA) [8]. Only five of the Organisation for Economic Co-operation and Development (OECD) member countries met this commitment in 2011 — Denmark, Luxembourg, the Netherlands, Norway, and Sweden [^9^]. So long as African governments fail to help themselves, it would appear that calls to wealthy nations will remain inconsequential.

Still, targets aside, the sub-Saharan share of the allocable donor assistance for health (DAH) has increased steadily to reach 56% in 2010 [7]. The $8.1b invested in sub-Saharan Africa that year is a testimony to donor responsiveness to the continent’s needs. It is however disconcerting to note that, in spite of the 2005 Paris Declaration on Aid Effectiveness and the Accra Agenda for Action, the share of DAH bypassing government and going to NGOs rose steadily from 3.1% in 1995 to 23.8% in 2004 and 66.7% in 2010 (calculated from statistics provided in Annex Table)[7]. In PEPFAR, the large U.S. government funded program that targets AIDS/HIV, only 17% of its budget has gone directly to its 31 partner governments [10].

This failure to support governments is undoubtedly the reason behind the discontent about progress in implementing the Paris Declaration on Aid Effectiveness expressed by the 56^th^ Session in Addis in 2006. The meeting noted that “even where robust sector plans and budgets existed, donors were reluctant to align with these plans or to provide flexible resources for implementation. There was general disillusionment with SWAps and multilateral development banks as acceptable instruments of support, and health sector expectations were seldom met due to the perpetuation of earmarked funds.”[11].”

This brief overview of what happened since Mexico confirms that African health budgets remain heavily reliant on outside sources of funding making their health systems extremely vulnerable to reductions in DAH. It is also clear that the low appreciation of the value of health research persists despite the declarations, making funding for research far more dependent on external sources than the total health budgets.

## Conclusion

The 21^st^ century began hopefully with growing African leadership in the health policy arena and an unprecedented surge in donor assistance for health. But after one decade it is clear that the current situation is not conducive to building strong national health research systems in Africa. Consequently, the promise of health systems strengthening may remain elusive, despite positive efforts. African countries are not acting according to their declarations, and are reneging on their commitment to take the lead by increasing their investments in health and research for health. Although international support for health has increased substantially in recent years, there has been a continued focus on disease-specific initiatives. Much donor support is funneled through international organizations, and country support continues to flow mainly to non-governmental organizations. The guidance of the Paris Declaration and the Accra Plan of Action are being royally ignored with more than two-thirds of donor assistance for health bypassing government.

These are the complexities that will await the outcome of work currently under way that is described in this supplement. A methodical framework for sustainability was adopted at the outset — partnerships worked closely with governments, the evidence base generated will be robust, and African leadership has been promoted. But the context in which these projects have been pursued suggests that a broader dialog on how international assistance for health is conceived will be needed to achieve results that can be scaleable and sustainable. Both African governments and donor countries will need to examine how they engage to improve health systems, a critical step in improving population health.

## List of abbreviations used

AHI: African Health Initiative; DAH: Donor assistance for health; DDCF: Doris Duke Charitable Foundation; MDGs: Millennium Development Goals; NGOs: Non-governmental organizations; OECD: Organisation for Economic Co-operation and Development; PEPFAR: President’s Emergency Plan for AIDS Relief; PHIT: Population Health Implementation and Training.

## Competing interests

The author serves as an Expert Advisor to the Doris Duke Charitable Foundation’s African Health Initiative.

## Declarations

This article has been published as part of *BMC Health Services Research* Volume 13 Supplement 2, 2013: Improving primary health care to achieve population impact: the African Health Initiative. The full contents of the supplement are available online at http://www.biomedcentral.com/bmchealthservres/supplements/13/S2. Publication of this supplement was supported by the African Health Initiative of the Doris Duke Charitable Foundation.
